# Effects of male telomeres on probability of paternity in sand lizards

**DOI:** 10.1098/rsbl.2018.0033

**Published:** 2018-08-22

**Authors:** Angela Pauliny, Emily Miller, Nicky Rollings, Erik Wapstra, Donald Blomqvist, Chris R. Friesen, Mats Olsson

**Affiliations:** 1 Department of Biological and Environmental Sciences, University of Gothenburg, Sweden; 2 School of Life Sciences, University of Sydney, Heydon-Laurence Building (A08), New South Wales 2006, Australia; 3 School of Biological Sciences, University of Tasmania, Hobart 7001, Australia; 4 School of Biological Sciences, The University of Wollongong, Australia

**Keywords:** telomeres, sperm competition, cryptic female choice, sand lizard (*Lacerta agilis*)

## Abstract

Standardized swim-up trials are used in *in vitro* fertilization clinics to select particularly motile spermatozoa in order to increase the probability of a successful fertilization. Such trials demonstrate that sperm with longer telomeres have higher motility and lower levels of DNA damage. Regardless of whether sperm motility, and successful swim-up to fertilization sites, is a direct or correlational effect of telomere length or DNA damage, covariation between telomere length and sperm performance predicts a relationship between telomere length and probability of paternity in sperm competition, a prediction that for ethical reasons cannot be tested on humans. Here, we test this prediction in sand lizards (*Lacerta agilis*) using experimental data from twice-mated females in a laboratory population, and telomere length in blood from the participating lizards. Female identity influenced paternity (while the mechanism was not identified), while relatively longer male telomeres predicted higher probability of paternity. We discuss potential mechanisms underpinning this result.

## 1. Introduction

Since Geoff Parker's demonstration of post-copulatory sexual selection and its impact on relative fitness, the overwhelming ubiquity of this process across taxa and traits has been repeatedly confirmed [1,2]. Much of the variation in a male's siring success has been attributed to the competitiveness of his ejaculate compared to rival males [1], but some of the variation in probability of paternity has also been ascribed to female characteristics affecting, and potentially biasing, male probability of paternity [2].

A technique sometimes used in *in vitro* fertilization technology are swim-up trials in which sperm traits or categories are identified that perform best under some standardized conditions [3]. Using such techniques in research on human spermatozoa, sperm more successful at standardized swim-up trials have longer telomeres (i.e. the non-coding chromosome ‘caps’ with several functions, such as protecting the coding chromosome parts at cell fission), and lower level of double DNA strand breakage [3]. Furthermore, recent work shows that telomere length correlates with several aspects of fertility (e.g. sperm numbers per ejaculate [4,5], and embryonic survival [4]). Thus, this predicts that sperm with longer telomeres should arrive more quickly, or often, at sites for fertilization in the female reproductive tract, resulting in higher siring success, something that for ethical reasons cannot be tested in humans. Here, we test the association between telomere length (in blood, as a proxy for sperm telomere length) and probability of paternity in the sand lizard, *Lacerta agilis*.

## 2. Material and methods

### (a) Multiple mating experiments

Wild-caught sand lizards were kept at facilities at the University of Gothenburg in the year 2000 for breeding experiments as outlined in [6–8]. Lizards were kept in individual cages (400 × 600 × 400 mm) containing a sand substrate and a flat rock over a moist patch of soil where females laid their eggs.

**Figure 1.**
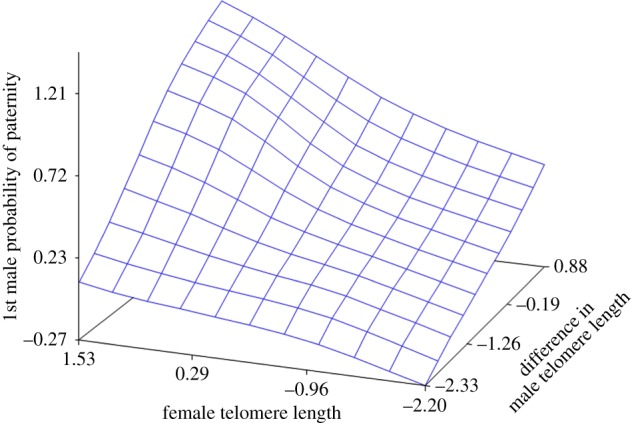
Response surface plot of first male probability of paternity plotted against female telomere length and the difference in male telomere lengths (male 1–male 2). (Online version in colour.)

For the experiments in this study, two males were selected at random and mated sequentially to a single female. Male mating order was recorded, although this has been demonstrated not to affect probability of paternity (figure 1) [6]. Males and females were weighed to the nearest 0.01 g and the consecutive number of a given copulation for a participating male was recorded to be able to control for sperm limitation. Once mated, females were kept separately at approximately 18°C with a 40 W spotlight at one end of each cage to enable females to bask, and thus attain body temperatures of up to 40°C if they chose to do so. Cages were checked twice daily for newly laid eggs, which were immediately removed and incubated (with one clutch per container) in moist vermiculite (1 : 10 water to vermiculite by volume) at 25°C.

### (b) Determination of paternity

The female, the two competing males and all hatchlings in a family were blood-sampled (hatchlings approx. 10 µl, adults approx. 50 µl) from the *vena angularis* (corner of the mouth). Samples were stored in 70% alcohol at −80°C until DNA extraction and molecular genetic analyses as described below. We genotyped individuals in multiplexed PCR reactions at up to 21 microsatellite loci using previously published protocols [9]. Paternity was determined based on the assignment of offspring alleles to one of the two competing males in a family. A male was excluded as the genetic father if he mismatched an offspring at more than one locus.

### (c) Telomere length assay

Relative telomere length (hereafter rTL) was measured using quantitative real-time PCR (qPCR [10]). Building on our previously described protocols (e.g. [11]), we have adapted the technique for use in sand lizards. Briefly, telomere repeats and glyceraldehyde-3-phosphate dehydrogenase (GAPDH) as reference gene were amplified from whole blood genomic DNA using previously published primers [12]. Each optimized reaction contained 1 ng DNA in a total volume of 10 µl 1× SsoAdvanced Universal SYBR Green Supermix (Bio-Rad). Final concentrations of forward and reverse primers for the telomere amplification were 100 and 200 nM, respectively, whereas 200 nM was used for each of the GAPDH primers. Amplifications were carried out on a Bio-Rad CFX96 qPCR machine, and reaction conditions included an initial denaturation at 96°C for 3 min, followed by 25 (telomere) or 40 (GAPDH) cycles of 96°C for 15 s and 56°C for 45 s. After each run was completed, a melt curve (55–96°C, 5 s hold time, 0.5°C increase cycle^−1^) was generated to confirm PCR specificity.

Experimental samples (randomly assigned to one of 12 plates) and an inter-plate calibrator (included on all plates) were analysed in triplicate, and average values were used in subsequent analyses. The intra-plate coefficient of variation (samples in triplicate) ranged between 0.0045 and 3.28% (telomere), and 0.016 and 2.47% (GAPDH), while the inter-plate coefficient of variation based on our calibrator sample was 3.72% (telomere), and 2.41% (GAPDH). We included a no template (negative) control in triplicate on each plate, none of which ever resulted in a fluorescent signal above the threshold. We estimated the amplification efficiency *E* from a standard curve, which consisted of five serial 1 : 5 dilutions of one sample loaded in triplicate on each plate (range 8–0.013 ng DNA per well). Standard curve characteristics and *E* of all plates are presented in the electronic supplementary material, file S1. To calculate rTL following Pfaffl [13], we used the average PCR efficiency for each assay (*n* = 12; telomeres, *E* = 2.0084 ± 0.0065; GAPDH, *E* = 2.039 ± 0.015).

### (d) Statistical analyses

In total, 92 offspring from 12 females, in 14 clutches, using 16 different males mated in random pair combinations were analysed. Hatchability in the examined clutches was 97% (±18, s.d.). One male pair combination was used twice but with the males in reversed order. Reuse of individual animals was driven by availability of animals in random combinations for different mating trials. There was no effect on probability of paternity of the difference in male body mass, number of copulations prior to the target copulation, which hemipenis a male used (left or right), or female mass (*p* > 0.279). These predictors were therefore not further analysed.

Mixed model logistic regression analyses were run in Proc GLIMMIX SAS 9.4 using siring success for the first male (sired/did not sire) as response variable (binomial distribution, logit link function). As predictors, we used the difference in male rTL (first male minus the second male trait value), and the rTL of the female. Female identity was used as random effect to control for sibling/half-sibling dependence and any female biasing of paternity, which has been demonstrated several times before in this species [14]. rTL was mean centred and standardized for plate number.

## 3. Results

We ran two models in Proc GLIMMIX, with (table 1) and without female identity number as a random effect. A significant female rTL effect on probability of paternity vanished with female number included in the model (female identity as random effect was significant, *χ*^2^ = 390, *p* < 0.01). The positive parameter estimate of the difference in male rTL demonstrates a higher probability of paternity for the first male the relatively longer telomeres he had (*p* = 0.014; table 1). The mean reproductive success of first males was 0.45 (±0.50, s.d.), whereas the corresponding effect for second males was 0.55 (±0.50, s.d.). Thus, a 95% confidence interval includes zero (1.96 × s.d.), which reconfirms our previous results of no mating order effect on probability of paternity [6].

**Table 1.** Proc GLIMMIX analysis in SAS 9.4 of the probability that a first male sires an offspring. (In this analysis, female number is included as a random effect, which is significant. Including female number as a random effect, renders female telomere length non-significant. rTL, relative telomere length. Log-likelihood ratio test of female number as random effect: *χ*^2^ = 390.0, *p* < 0.01.)

solutions for fixed effects					
effect	estimate	standard error	d.f.	*t*	Pr >|*t*|
intercept	2.3741	1.3304	10	1.78	0.1047
female rTL	1.2987	0.8510	79	1.53	0.1310
male rTL difference	2.0598	0.8218	79	2.51	0.0142

## 4. Discussion

In the present study, we find that males with longer telomeres had a higher probability of siring offspring, while controlling for female identity in the model. Previous work has shown that male and female relatedness influences a male's probability of paternity, and that more closely related males have similar siring success [14]. None of these results are easily reconciled by straightforward, numerical ‘raffles’ effects on probability of paternity (i.e. higher siring success from producing more spermatozoa), although such processes are likely to occur also in this species [6]. The—admittedly unidentified—mechanism seems much more likely to be a genetic effect on fertilization. In a recent publication, we review the potential links between telomeres and relative fitness and find more support for correlational effects than directly causal ones; the magic bullet of telomere effects on fitness are still to be demonstrated [15]. The links between genetic effects and the telomere characteristics associated with siring success could be several: (i) the correlation between telomere length and DNA damage in human sperm swim-up trials may simply indicate that telomere length is an indicator of overall, diploid genetic quality (e.g. level of heterozygosity at the major histocompatibility complex); (ii) it is well known that sperm performance declines with senescence. Thus, if telomeres reflect biological ageing, a correlation between telomere attrition and sperm performance is intuitive; (iii) another possibility is that telomeres have some active role in affecting probability of fertilization *per se*. For example, females (and eggs) should be under selection to identify and destroy (or reject) ‘poor sperm’ that fertilize eggs but produce inferior offspring; and (iv) telomeres correlate with some other aspect of genetic or phenotypic quality on which a cryptic female choice is based. For all these processes, telomere length in sperm and blood needs to be correlated, a piece of information that we presently do not have access to in sand lizards. However, research on humans suggest that telomere length in blood and sperm are highly correlated. Thus, to summarise, telomere length predicts paternity in patterns challenging to a simplistic understanding of post-copulatory mechanisms.

## Supplementary Material

Sperm Comp Telomere Supplementary File 1

## Data accessibility

Data have been submitted in an electronic supplementary material.

## References

[1] Parker GA 1970 Sperm competition and its evolutionary consequences in the insects. Biol. Rev. 45, 525–567. (10.1111/j.1469-185x.1970.tb01176.x)

[2] Eberhard WG 1996 Female control: sexual selection by cryptic female choice. Princeton, NJ: Princeton University Press.

[3] Santiso R, Tamayo M, Gosálvez J, Meseguer M, Garrido N, Fernández JL 2010 Swim-up procedure selects spermatozoa with longer telomere length. Mutat. Res.-Fundam. Mol. Mech. Mutagen. 688, 88–90. (10.1016/j.mrfmmm.2010.03.003) 20226199

[4] Cariati F, Jaroudi S, Alfarawati S, Raberi A, Alviggi C, Pivonello R, Wells D 2016 Investigation of sperm telomere length as a potential marker of paternal genome integrity and semen quality. Reprod. Biomed. 33, 404–411. 10.1016/j.rbmo.2016.06.006 27396673

[5] Ferlin A, Rampazzo E, Rocca MS, Keppel S, Frigo AC, De Rossi A, Foresta C 2013 In young men sperm telomere length is related to sperm number and parental age. Hum. Reprod. 28, 3370–3376. 24166593 10.1093/humrep/det392

[6] Olsson M, Gullberg A, Tegelström H 1994 Sperm competition in the sand lizard, *Lacerta agilis*. Anim. Behav. 48, 193–200. (10.1006/anbe.1994.1226)

[7] Olsson M, Ujvari B, Madsen T, Uller T, Wapstra E 2004 Haldane rules: costs of outbreeding at production of daughters in sand lizards. Ecol. Lett. 7, 924–928. (10.1111/j.1461-0248.2004.00652.x)

[8] Olsson M, Loeb L, Lindsay W, Wapstra E, Fitzpatrick L, Shine R. 2018 Extreme plasticity in reproductive biology of an oviparous lizard. Ecol. Evol. 8, 6384–6389. (10.1002/ece3.4247) 30038742 PMC6053574

[9] Schwartz TS, Olsson M 2006 Microsatellite markers developed for a Swedish population of sand lizard (*Lacerta agilis*). Conserv. Genet. 9, 715–717. (10.1007/s10592-006-9238-2)

[10] Cawthon RM 2002 Telomere measurement by quantitative PCR. Nucleic Acids Res. 30, 47 (10.1093/nar/30.10.e47) 12000852 PMC115301

[11] Johnsen A, Pauliny A, Lifjeld JT, Blomqvist D 2017 Is telomere length associated with mate choice in a songbird with a high rate of extra-pair paternity? PLoS ONE 12, e0182446 (10.1371/journal.pone.0182446) 28783753 PMC5544213

[12] Criscuolo F, Bize P, Nasir L, Metcalfe NB, Foote CG, Griffiths K, Gault EA, Monaghan P 2009 Real-time quantitative PCR assay for measurement of avian telomeres. J. Avian Biol. 40, 342–347. (10.1111/j.1600-048x.2008.04623.x)

[13] Pfaffl MW 2001 A new mathematical model for relative quantification in real-time RT-PCR. Nucleic Acids Res. 29, e45 (10.1093/nar/29.9.e45) 11328886 PMC55695

[14] Olsson M, Shine R, Madsen T, Gullberg A, Tegelström H 1997 Sperm choice by females. Trends Ecol. Evol. 12, 445–446. (10.1016/s0169-5347(97)85751-5) 21238151

[15] Olsson M, Friesen CR, Wapstra E 2018 Ectothermic telomeres: it's time they came in from the cold. Phil. Trans. R. Soc. B 373, 20160449 (10.1098/rstb.2016.0449) 29335373 PMC5784069

