# Supplementary material for: Effects of male telomeres on probability of paternity in sand lizards
Source: Biol Lett. 2018 Aug 22;14(8):20180033. doi: 10.1098/rsbl.2018.0033 (PMC6127112; doi:10.1098/rsbl.2018.0033)
Supplement: Sperm Comp Telomere Supplementary File 1 [file rsbl20180033supp1.docx]

**Supplementary file 1**

Summary of standard curve characteristics for telomere and GAPDH qPCR assays.

On each of 12 telomere and 12 GAPDH qPCR plates, a standard curve was included in triplicate. For each assay separately, the mean ± s.e. (first rows), as well as the range (second rows) are presented for the slope, y-intercept, coefficient of determination R^2^, and PCR efficiency E.

|  | Telomere | GAPDH |
| --- | --- | --- |
| Slope | -3.30 ± 0.015 | -3.24 ± 0.033 |
|  | -3.39 – -3.20 | -3.38 – -3.028 |
| y-intercept | 10.34 ± 0.064 | 28.66 ± 0.10 |
|  | 9.99 – 10.75 | 28.053 – 29.32 |
| R^2^ | 0.996 ± 0.000287 | 0.992 ± 0.00104 |
|  | 0.995 – 0.997 | 0.983 – 0.995 |
| PCR efficiency | 2.0084 ± 0.0065 | 2.039 ± 0.015 |
|  | 1.97 – 2.052 | 1.98 – 2.14 |
